# Inhibition of norepinephrine signaling during a sensitive period disrupts locus coeruleus circuitry and emotional behaviors in adulthood

**DOI:** 10.1038/s41598-023-29175-x

**Published:** 2023-02-22

**Authors:** Qingyuan Meng, Alvaro L. Garcia-Garcia, Alex Dranovsky, E. David Leonardo

**Affiliations:** Dranovsky-Leonardo Lab (ADL Lab), Department of Psychiatry, Division of Integrative Neuroscience, Columbia University and the New York State Psychiatric Institute, 1051 Riverside Dr. Box 87, New York, NY 10032 USA

**Keywords:** Neural circuits, Stress and resilience, Emotion

## Abstract

Deficits in arousal and stress responsiveness are a feature of numerous psychiatric disorders including depression and anxiety. Arousal is supported by norepinephrine (NE) released from specialized brainstem nuclei, including the locus coeruleus (LC) neurons into cortical and limbic areas. During development, the NE system matures in concert with increased exploration of the animal’s environment. While several psychiatric medications target the NE system, the possibility that its modulation during discreet developmental periods can have long-lasting consequences has not yet been explored. We used a chemogenetic strategy in mice to reversibly inhibit NE signaling during brief developmental periods and then evaluated any long-lasting impact of our intervention on adult NE circuit function and on emotional behavior. We also tested whether developmental exposure to the α2 receptor agonist guanfacine, which is commonly used in the pediatric population and is not contraindicated during pregnancy and nursing, recapitulates the effect seen with the chemogenetic strategy. Our results reveal that postnatal days 10–21 constitute a sensitive period during which alterations in NE signaling lead to changes in baseline anxiety, increased anhedonia, and passive coping behaviors in adulthood. Disruption of NE signaling during this sensitive period also caused altered LC autoreceptor function, along with circuit specific changes in LC-NE target regions at baseline, and in response to stress. Our findings indicate an early critical role for NE in sculpting brain circuits that support adult emotional function. Interfering with this role by guanfacine and similar clinically used drugs can have lasting implications for mental health.

## Introduction

The central noradrenergic system, comprised of several small densely packed brainstem nuclei that supply noradrenaline throughout the brain^1^, is involved in a wide range of normal behavioral and physiological responses such as attention, vigilance and arousal^2–5^, and is a major component of the centrally mediated stress response^6,7^. Behaviors supported by normal NE function are disrupted in numerous psychiatric disorders^8^. Accordingly, dysregulation within the NE system has been implicated in the pathophysiology and treatment of anxiety and depression^9^. For example, agonists and antagonists of the Gi coupled α2 receptor alleviate or exacerbate anxiety symptoms respectively^10^. Moreover, fewer NE transporters (NET) have been reported in the LC of post-mortem brain tissue from depressed patients^11,12^. Together, this evidence implicates noradrenergic dysfunction in the biology of neuropsychiatric disorders.

While it is now clear that noradrenergic projections arising from brainstem adrenergic nuclei that are more heterogeneous than previously thought^1^, studies examining the developmental function of noradrenaline on the forebrain have focused on the LC, long thought to be the major source of NE to this area. For example, studies in rats show that the ability of NE to modulate brain functions develops postnatally as the maturing LC begins to exhibit activity in response to environmental changes^13^. For example, NE signaling is critical for early life sensory and odor-based attachment learning^14,15^. These processes are complete by postnatal day 10–15 (P10–P15), just as LC autoinhibition through α2A autoreceptors comes online^14,16^. Interestingly, the time period that follows (P15–P21) coincides with the emergence of normal exploration and habituation in rodents^17^. These and other milestones of LC function occur in the context of steadily increasing NE from birth until P30–40 when adult concentrations are reached^18–20^. Genetic models in mice also demonstrate that disrupting NE signaling has developmentally distinct consequences. While α2A receptor KO mice display an anxiety and depression-like phenotype^21,22^, adult suppression of LC α2A autoreceptors in rats leads to the opposite outcome^23,24^. Moreover, drugs that target the NET for the treatment of depression are ineffective in juveniles but highly effective in adults^25–27^, suggesting that the effects of modulating NE signaling on emotion related circuits differ across the lifespan. Thus, given that NE has distinct roles in circuit maturation, we hypothesized that briefly disrupting NE function during different developmental periods would have distinct and lasting consequences.

Here, we tested whether disruption of normal NE signaling during brief developmental time periods has long-term consequences for normal adult NE function in the brain. We found that chemogenetic inhibition of NE signaling during P10–P21, but not during other periods, resulted in persistent molecular adaptations within the NE system along with a depression-like phenotype with some anxiety features. Administration of the α2A agonist guanfacine, a medication used to treat blood pressure and attention deficit hyperactivity disorder, during this sensitive period phenocopied the chemogenetic results, highlighting the potential vulnerability of the developing NE brain system to psychoactive medications.

## Results

### Manipulation of NE neuronal activity via conditional expression of DREADDs in mice

**Figure 1:**
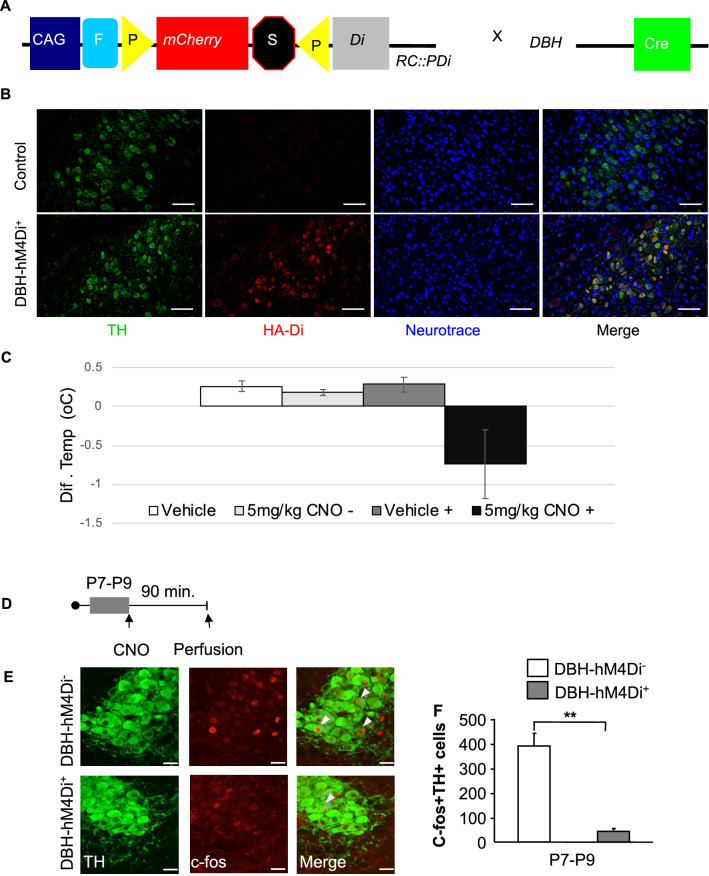
CNO interferes with LC function in DBH-hM4Di mice. (**A**) The inhibitory DREADD hM4Di (Di) was expressed in NE neurons by crossing the RC::PDi (hM4Di) line with a dopamine β-hydroxylase cre line (DBH-Cre), resulting in a Rc:PDi;DBH-cre (DBH-hM4Di^+^) line that expresses hM4Di in NE cells off of a CAG promoter. (**B**) Immunostaining for TH and HA-tag, and labelling with Neurotrace in the LC of DBH-hM4Di^+^ animals confirm the specificity of hm4Di expression in NE neurons. Scale bars = 100 μm. (**C**) Average change in temperature over the course of 60 min (sampled every 10 min) to an injection of vehicle or 5 mg/kg of CNO in DBH-hM4Di^−^ and DBH-hM4Di^+^ mice in adulthood. Temperature is shown as change from baseline. (**D**–**F**) Chemogenetic inhibition of NE neurons at P7–P9. (**D**) Experimental timeline. (**E**) Representative images of c-fos and TH double immunostaining in the LC after a single CNO i.p. injection (5 mg/kg) in P7–P9 DBH-hM4Di(−) and (+) mice. Single white arrows depict double-labeled cells. Scale bars represent 10 μm. (**F**) decrease in the number of TH + cells that are also c-fos + in P7–P9 DBH-hM4Di + when compared to their DBH-hM4Di− control mice. Means are represented as ± SEM. (*p < 0.05; **p < 0.01).

To interfere with NE signaling in vivo, we crossed RC::PDi mice^28^ to a dopamine β-hydroxylase cre line (DBH-Cre)^29^. The resulting DBH-cre;RC:PDi (DBH-hM4Di^+^) mice express hemaglutinin (HA) tagged hM4Di (inhibitory DREADD) in NE neurons (Fig. 1A,B). Immunostaining in the LC against Tyrosine hydroxylase (TH) and HA confirmed highly selective hM4Di expression in NE synthesizing neurons (Fig. 1B). This approach selectively renders NE neurons more resistant to activation whenever hM4Di is stimulated by the synthetic ligand clozapine-n-oxide (CNO)^30^.

We initially validated our approach taking advantage of the fact that α2-A receptor agonists cause a modest decrease in body temperature in adult animals by inhibiting NE neurons^31^. We thus tested whether a CNO injection would also cause a decrease in body temperature by inhibiting NE neurons. Indeed, administration of CNO (5 mg/kg) but not vehicle resulted in a decrease in body temperature in adult DBH-hM4Di^+^ mice. No decrease was seen in DBH-hM4Di^-^ mice treated with vehicle or CNO (genotype × treatment interaction: F_1,16_ = 51.65, p < 0.0001; post hoc hM4Di + p = 0.001, hM4Di− p = 0.89; n = 5/group) (Fig. 1C). This result demonstrates that functional hM4Di receptors are expressed in NE neurons of adult animals.

To ensure that CNO mediated hM4Di signaling could be mobilized early in development, we injected DBH-hM4Di^+^ and control (DBH-hM4Di^−^) P7–P9 pups with CNO in their home cage and evaluated c-fos expression in the LC 90 min later (Fig. 1D,E). At this age, LC neurons fire in response to external stimuli and we hypothesized that handling and injecting the mice would activate noradrenergic cells. DBH-hM4Di^+^ mice had fewer c-fos + cells overall (Supplementary Fig. S2), fewer c-fos positive cells within the TH positive pool (t_(5)_ = 3.725, p = 0.01; n = 3–4/group) (Fig. 1F), with no change in the overall number of NE cells (TH+) (Supplementary Fig. S2). These results demonstrate that the chemogenetic intervention can attenuate noradrenergic neuronal activity as early as the end of the first post-natal week.

### Repeated chemogenetic inhibition of NE neurons between P2 and P21 results in increased anxiety in the open-field and depression-like behavioral phenotype later in life

**Figure 2:**
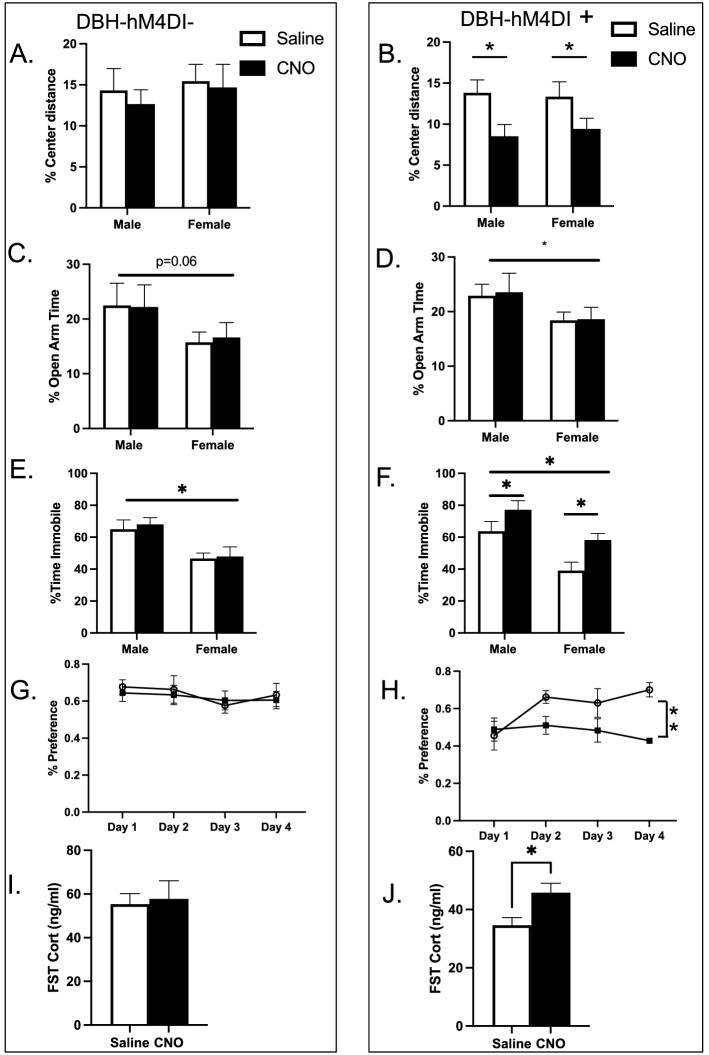
Enhanced anxiety and depression-like behavior in adult male and female mice after chemogenetic inhibition of NE neurons between P2–P21. Open Field (**A**,**B**): (**A**) No main effect of CNO treatment in hM4Di—mice on percentage center distance in the open-field when compared to vehicle controls. No effect of sex. (**B**) Main effect of CNO treatment in hM4Di + animals on percent center distance in the open field when compared to controls. No effect of sex. Elevated Plus Maze (**C**,**D**): (**C**) No main effect of CNO treatment detected in hM4Di− mice on % open arm time when compared to vehicle controls. Effect of sex, p = 0.06. (**D**) No main effect of CNO treatment detected in hM4Di + mice on % open arm time when compared to vehicle controls. Main effect of sex detected. Forced Swim Test (**E**,**F**): (**E**) No main effect of CNO treatment on % time immobile detected in hM4Di− mice when compared to vehicle controls. Main effect of sex detected. (**F**) Main effect of CNO treatment and main effect of sex detected in hM4Di + mice on percent time immobile when compared to vehicle controls. Sucrose Preference Test (**G**,**H**): (**G**) No difference in sucrose preference across 4 test days in CNO treated hM4Di- mice when compared to controls. (**H**) Main effect of CNO treatment in hM4Di + mice on sucrose preference across 4 test days when compared to controls. Forced swim-stress induced CORT (**I**,**J**): (**I**) No effect of CNO treatment detected on FST induced CORT in hM4Di− mice. (**J**) Increased CORT in CNO treated hM4Di + mice after FST when compared to vehicle treated controls. Means (**A**–**F**) were compared using two-way ANOVA, (**G**,**H**) were compared with mixed model ANOVA and (**I**,**J**) with t-Test. Means are represented as ± SEM. (*p < 0.05; **p < 0.01).

We hypothesized that normal NE function in early development is critical to establish later behavioral responses and initially selected virtually the entire pre-weaning (P2–P21) for daily treatment with CNO (5 mg/kg). To ensure that the CNO intervention was specific only to DREADD expressing animals, we exposed both a hM4Di + and hM4Di− cohort to daily administration of either CNO or saline from P2–P21. After the treatment period, animals were allowed to develop normally until adulthood when measures of anxiety were assessed in the open field and the elevated plus maze. As expected, in the hM4Di− animals, there were no differences in percent distance in the center in the open field between CNO treated animals and controls (hM4Di− treatment F_1,37_ = 0.257, p = 0.62; or sex: F_1,37_ = 0.428, p = 0.79; n = 9–14/group) (Fig. 2A). However, in the hM4Di + animals there was a robust effect of CNO treatment (hm4Di + : treatment F_1,42_ = 9.185, p < 0.01; sex: F_1,42_ = 0.0174, p = 0.9; n = 9–14/group)(Fig. 2B). No effect of sex was observed in either the hM4Di + or hM4Di– cohorts in this test. Interestingly, in the elevated plus maze, no effect of CNO treatment was observed in percent time spent in the open arms in hM4Di− group (hM4Di−: treatment F_1,38_ = 0.009, p = 0.93; sex: F_1,38_ = 3.816, p = 0.06; n = 9–14/group) (Fig. 2C) nor in hM4Di + group (hM4Di + : treatment F_1,42_ = 0.033, p = 0.86 sex: F_1,42_ = 4.137, p < 0.05; n = 9–13/group) (Fig. 2D). Evidence for a main effect of sex was detected in the hM4Di + group, with a similar pattern, that missed significance in the hM4Di− group.

Next, we examined the effect of developmental suppression of NE neurons on active coping behavior in the forced-swim test (FST) in adulthood. No main effect of CNO treatment was observed in percent time immobile in the hM4Di- group (hM4Di−: treatment F_1,36_ = 0.194, p = 0.66 sex: F_1,36_ = 14.88, p < 0.001; n = 9–13/group) (Fig. 2E), while a main effect of CNO treatment was observed in the hM4Di + group (hM4Di + : treatment F_1,42_ = 9.268, p < 0.01 sex: F_1,42_ = 16.71, p < 0.001; n = 9–13/group) (Fig. 2F). Both hM4Di− and hM4Di + groups revealed a main effect of sex.

We then tested the mice in the sucrose preference test, which measures anhedonia, a core feature of depression^32^. We detected no difference in sucrose preference across 4 test days in CNO treated hM4Di− mice when compared to controls (hM4Di−: treatment F_1,19_ = 0.34, p = 0.95, n = 9–10 males/group) (Fig. 2G), while a significant main effect of CNO treatment and time × treatment interaction was observed in the hM4Di + group (hM4Di + : treatment F_1,10_ = 12.91, p < 0.01, time × treatment interaction F_3,25_ = 3.056, p ≤ 0.05, n = 9–10 males/group) (Fig. 2H).

Given the role of the NE system in mediating stress responses and in depression, we decided to test the hypothalamic–pituitary–adrenal axis reactivity in these mice. We used a forced swim stressor to elicit corticosterone (CORT) responses and collected blood samples shortly thereafter. As expected, no effect of CNO treatment was observed in hM4Di− animals (hM4Di−: t_(12)_ = 0.81, p = 0.81, n = 7–10 males/group), while an effect of CNO treatment was seen in hM4Di + animals (hM4Di + : t = 2.567, df = 16, P = 0.02, n = 7–10 males/group).

In the absence of the hM4Di DREADD, CNO treatment had no independent effect, demonstrating that all behavioral effects observed were due to CNO acting through the hM4Di DREADD. We also found that despite some sex differences in baseline behavior, there were similar effects of the DREADD intervention on male and female mice in the open field, elevated plus and forced swim tests, with no evidence of any treatment × sex interactions. As a result of these findings, further testing was conducted in hM4Di + male cohorts.

### Repeated chemogenetic inhibition of NE neurons between P10 and P21, but not other developmental time windows results in increased anxiety in the open-field and depression-like behavioral phenotype later in life

**Figure 3:**
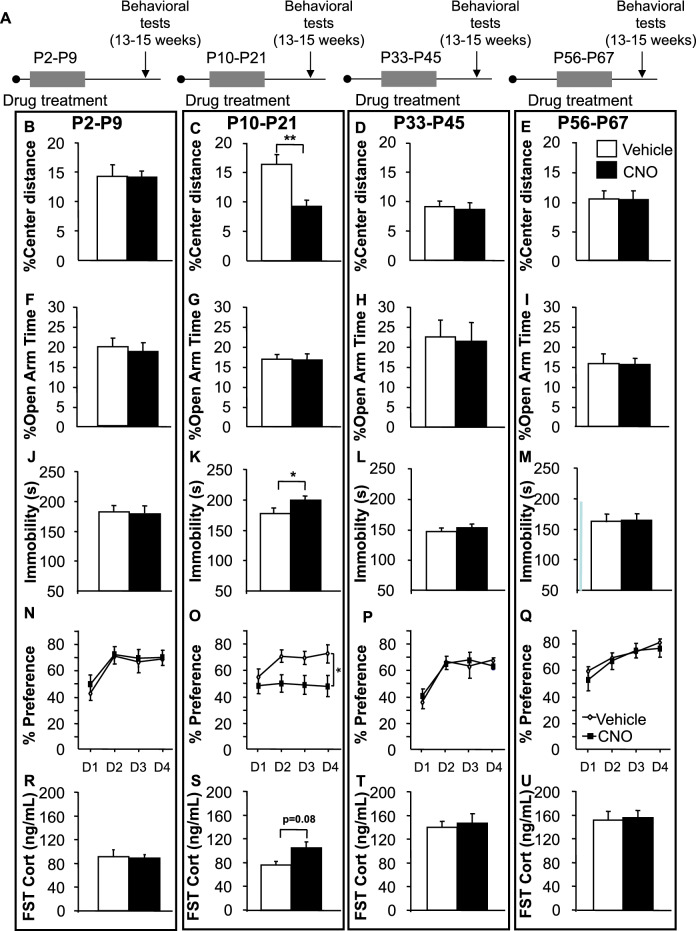
Enhanced anxiety and depression-like behavior in adult mice after chemogenetic inhibition of NE neurons between P10–P21 but not after P2–P9 or P33–P44 or P56–P67. (**A**) Experimental timeline. (**B**–**E**) Decreased percentage center distance in the open-field after P10–P21 intervention but not after P2–9, P33–P44 or P56–P67. (**F–I**) No differences were detected in time spent in the open arms in the elevated-plus maze. (**J–M**) Immobility in the FST: Differences were observed in the P10–P21, but not other groups when comparing CNO to vehicle mice. (**N–Q**) Sucrose Preference test. Differences in preference were observed in the P10–P21 group but not others when comparing CNO to vehicle treatment during testing days (Day 1–4). (**R**–**U**) Forced swim-stress induced CORT. CNO treated group compared to vehicle treated mice. Means were compared using two-way ANOVA. Post hoc test compare vehicle vs CNO treated animals within each age group. Means are represented as ± SEM. (*p < 0.05; **p < 0.01). See also Supplementary Fig. S1A, S2 and S3.

Having identified behavioral effects of disrupting noradrenergic signaling throughout the pre-weaning period, we endeavored to test whether we could identify a more discrete pre-weaning period that would be sensitive to disruption. We further set out to test whether the pre-weaning period was special or whether the noradrenergic system was sensitive to disruption during other, later developmental time periods. Although the transgenic system we exploited affected all noradrenergic neurons, we hypothesized that the behavioral effects were likely due to effects on the LC and thus set out to test discrete postnatal developmental periods related to LC maturation (Fig. 3A and Supplementary Fig. S1A). The LC has little spontaneous activity in the first week of life, but bursts of activity can be elicited by external stimulation^13^. Between P10 and weaning (P21), robust spontaneous LC firing emerges and α2A receptor negative feedback is established^33^. However, adult NE levels are not reached until adolescence, during the fifth week of life^16^. We therefore selected P2–P9, P10–21, P33–P44, and an adult reference period (P56–P67) for subsequent experiments.

As before, we treated each cohort with either CNO (5 mg/kg) or vehicle during the proscribed time-period. The cohorts were then allowed to age normally until 13–15 weeks of age when behavioral testing began (Fig. 3A). We employed the same series of tests described for the P2–P21 cohort to assess exploration, anxiety, passive coping, sucrose preference, and HPA axis reactivity to the forced swim test. In the open field, adult DBH-hM4Di^+^ mice treated with CNO during P10–P21 but not during the other time periods, displayed decreased percent distance traveled in the center (treatment F_(1,132)_ = 3.595, p = 0.06; treatment age F_(1,132)_ = 5.633; treatment age × treatment F_(1,132)_ = 3.429, p = 0.02; post hoc: P2–P9, p > 0.99; P10–P21, p < 0.001; P33–P44, p = 0.99; P56–P67, p > 0.99; n = 15–20/group) (Fig. 3B–E). Importantly, general activity was not affected as we did not detect significant changes in total path traveled (Supplementary Fig. S3A–C). In the elevated-plus maze no significant differences were detected between groups in percent time spent in the open arms (treatment F_(1,131)_ = 0.027, p = 0.87; treatment age F_(1,131)_ = 1.933, p = 0.13; n = 15–20/group) (Fig. 3F–I).

In the forced-swim test (FST), of the four time periods tested, only DBH-hM4Di^+^ mice treated with CNO during P10–P21 displayed increased immobility (treatment F_(1,136)_ = 2.211, p = 0.14; treatment age F_(3,136)_ = 12.74, p < 0.001; post-hoc P2–P9, p = 0.99; P10–P21, p = 0.02; P33–P44, p = 0.98; P56–P67, p = 0.99; n = 15–20/group) (Fig. 3J–M).

In the sucrose preference test, once again, only the P10–P21 cohort treated with CNO exhibited differences from vehicle treated animals during the choice days (Days 1–4) (treatment F_(1,120)_ = 1.414, p = 0.23; treatment age F_(3,120)_ = 2.397, p = 0.07, age × treatment interaction F_(3,120)_ = 2.662, p = 0.05: post hoc : P2–P9, p = 0.90; P10–P21, p = 0.02; P33–P44, p = 0.99; P56–P67, p = 0.99; n = 14–20/group) (Fig. 3N–Q). These results are remarkably consistent with those obtained in the P2–P21 cohort and indicate that inhibition of NE neurons during P10–P21, but not before (P2–P9) or during the later time windows tested, is necessary to generate the increased anxiety in the open-field test, passive coping in the FST and decreased sucrose preference, observed.

Finally, as before, we used a forced swim stressor to elicit corticosterone (CORT) responses and collected blood samples shortly after. Consistent with all of our other results, only the P10–P21 CNO treated group displayed exaggerated stress-induced CORT from its respective controls although this difference did not survive multiple correction (treatment F_(1,68)_ = 2.004 p = 0.16; treatment age F_(3,68)_ = 22.33, p < 0.001; treatment age × treatment F_(3,68)_ = 1.207, p = 0.31: post hoc: P2–P9:, p > 0.99; P10–P21: p = 0.08; P33–P44: p = 0.99; P56–P67: p = 0.99; n = 9–11/group) (Fig. 3R–U). Importantly, no differences in baseline CORT levels were detected during the diurnal cycle (Supplementary Fig. S3E–H).

Taken together, our results indicate that the P10–P21 period, when LC neurons are acquiring spontaneous firing and developing negative feedback, is sensitive to disruption in a way that has significant consequences to behavioral responses later in life. No such effects are seen when NE signaling is disrupted earlier, or in late adolescence or adulthood.

### Transient inhibition of NE neurons between P10–P21 has long lasting effects on stress-induced LC-NE neuronal activation

**Figure 4:**
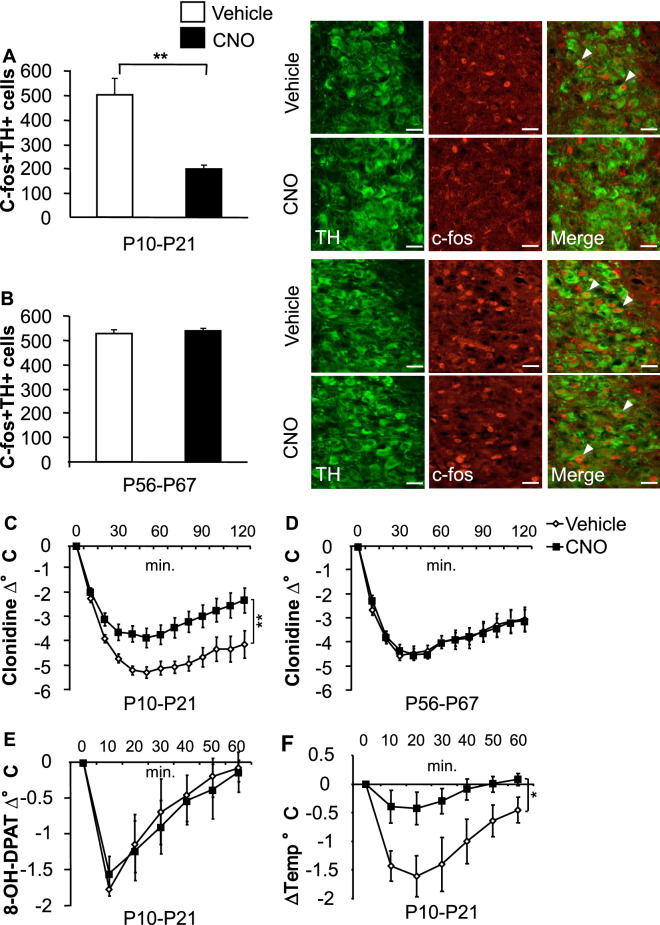
Chemogenetic inhibition of NE neurons between P10–21 has long-term consequences for stress-induced LC-NE reactivity and clonidine induced hypothermia. (**A**,**B**) c-fos and TH double immunostaining in the LC after a forced swim stressor in adult DBH-hM4Di^+^ mice that had previously been treated with CNO and vehicle during different developmental time periods. Single white arrows depict double-labeled cells. Scale bars represent 20 µm. Decrease in the number of TH + cells that are also c-fos + in the P10–P21 (**A**) but not in the P56–P67 group (**B**). (**C**,**D**) Decreased hypothermic response to clonidine in P10–P21 (**C**) but not in the P56–P67 group (**D**). (**E**) No changes were observed in 8-OH-DPAT (5-HT_1A_ agonist) induced hypothermia in the P10–P21 group. (**F**) Hypothermic response to intra-LC infusion of clonidine but not vehicle in the P10–21 group. Means are represented as ± SEM. *p < 0.05; **p < 0.01). See also Supplementary Fig. S4.

Having observed that interfering with normal NE signaling during the P10–P21 time-period results in sustained changes in behavioral and physiological reactivity, we tested whether we could detect sustained changes in the response properties of central NE neurons. To do so, we chose to evaluate c-fos expression in the LC of adult animals in response to a forced-swim stressor. Specifically, we compared mice that were treated during the developmental P10–P21 time window to an adult control group that had been treated from P56–P67. We detected fewer c-fos positive cells amongst NE (TH +) cells in the LC of adult mice that were treated with CNO during P10–P21 but not during the adult time window (P56–P67) when compared to their respective controls (treatment F_(1,15)_ = 10.5, p < 0.01; treatment age F_(1,15)_ = 6.12, p = 0.03, treatment age × treatment interaction F_(1,15)_ = 13.15, p < 0.01; post hoc P10–P21, p < 0.001; P56–P67 p = 0.95; n = 4–5/group) (Fig. 4A,B and Supplementary Fig. S4A,D). No difference in the number of c-fos positive cells in TH negative cells was detected in any of the groups (Supplementary Fig S4C,F). Further, the number of TH positive neurons remained unchanged (Supplementary Fig. S5B,E). Thus, NE signaling inhibition during P10–P21 leads to a decreased stress-induced activation of LC-NE cells in adulthood, without impacting their number. Whether other central NE populations are similarly affected was not examined.

### Inhibition of NE neurons between P10–P21 interferes with α2-A mediated thermoregulation in adults

α2-A receptors are the predominant autoreceptors on LC-NE neurons that provide negative feedback to the LC^34^. To assess α2-A receptor function in adulthood, mice were acutely treated with the α2-A receptor agonist clonidine (0.5 mg/kg), and their core body temperature was measured. Interestingly, mice treated with CNO during P10–P21, displayed an attenuated drop in temperature when compared to their controls (treatment F_(1,17)_ = 9.227, p < 0.01; time F_(1.874,31.86)_ = 70.64, p < 0.001; treatment × time interaction F_(12,204)_ = 4.083, p < 0.001; n = 9–10/group) (Fig. 4C) while the P56–67 CNO treated groups drop in temperature over time did not differ from their controls (treatment F_(1,18)_ = 0.007, p = 0.93; time F_(1.961,35.29)_ = 48.93, p < 0.001; treatment × time interaction F_12,216_ = 0.1922, P = 0.99; n = 10/group) (Fig. 4D). Next, to ensure that the observed changes were not the result of a general deficit in thermoregulation, we injected the 5-HT_1A_ agonist 8-OH-DPAT, as 5-HT_1A_ agonists are well known to decrease body temperature^28^. This elicited an acute drop in temperature with no differences observed between CNO and vehicle groups (treatment: F_(1,8)_ = 0.179, p = 0.68; time F_(2.448,19.58)_ = 32.29, p < 0.001, treatment × time interaction F_(6,48)_ = 0.88, p = 0.52; n = 5/group) (Fig. 4E). Finally, to confirm that the hypothermic effects were mediated in part through the LC, we injected vehicle or clonidine directly into the LC. Vehicle injected animals showed minimal change from baseline while clonidine injected animals showed a significant hypothermic effect (treatment: F_(1,14)_ = 6.780, p = 0.02; time F_(2.0.167,30.34)_ = 30.34, p < 0.001, treatment × time interaction F_(6,84)_ = 4.08, p =  < 0.01; n = 7–9/group) (Fig. 4F). These results indicate that interfering with NE activity during P10–P21, but not during P56–P67 results in an attenuation of the response to the α2-A agonist clonidine.

### Inhibition of NE neurons between P10–P21 leads to long-term molecular adaptations in the adult LC-NE circuit

**Figure 5:**
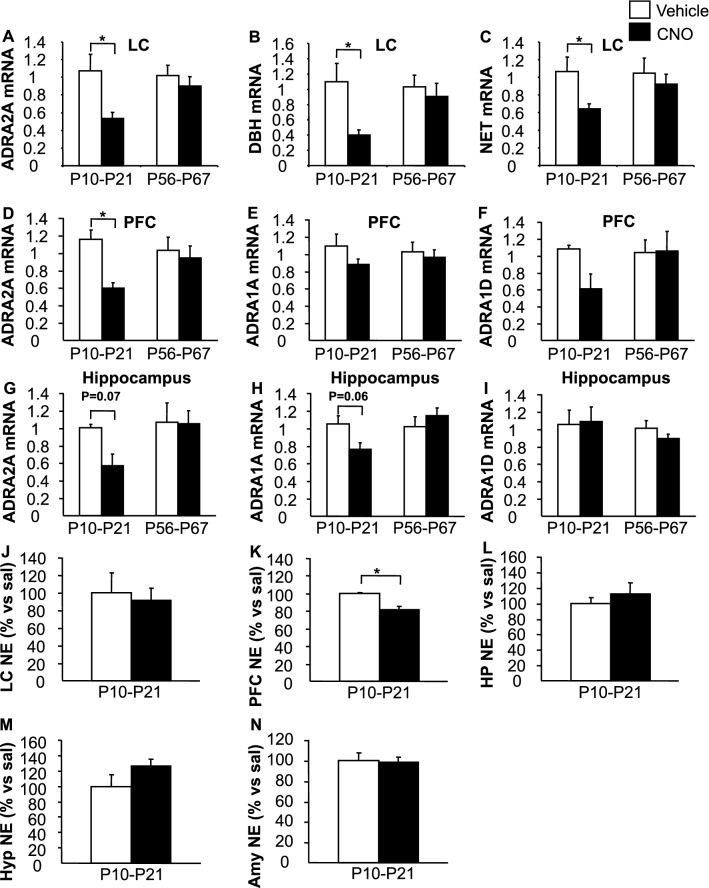
Selective LC-NE system adaptations after chemogenetic inhibition of NE neurons (**A**–**C**) CNO treatment of DBH-hM4Di^+^ mice between P10–21, but not P56–P67, results in decreased LC (**A**) ADRA2A (**B**) DBH and (**C**) NET mRNA expression in the LC. **(D**–**F)** CNO treatment of DBH-hM4Di^+^ mice between P10–21, but not P56–P67, results in decreased (**D**) PFC ADRA2A. Neither P10–P21 nor P56–P67 treatment affects PFC mRNA levels for ADRA1A (**E**) or ADRA1D. (**G**–**I**) CNO treatment of DBH-hM4Di^+^ mice between P10–21, but not P56–P67, results in lower (**G**) hippocampal ADRA2A and ADRA1A (**H**) along with no changes in ADR1D mRNA levels in adulthood. (**J**) No differences were detected in NE levels in the LC in mice treated with CNO during P10–P21 when comparing them to vehicle controls. (**K**) In contrast, P10–P21 CNO treatment results in decreased NE levels in the PFC in adulthood when compared to vehicle controls. (**L)** No changes were detected in adult NE levels after P10–P21 CNO intervention in the hippocampus. (**M**) No changes were detected in adult NE levels after P10–P21 CNO intervention in the hypothalamus. (**N**) No changes were detected in adult NE levels after P10–P21 CNO intervention in the amygdala. Means (**A**–**I**) were compared using two-way ANOVA and (**J**–**N**) with one-way ANOVA. Means are represented as ± SEM. (*p < 0.05; **p < 0.01). See also Supplementary Figs. S5 and S6.

Identification of physiological changes in response to an α2-A agonist in the P10–P21 cohort suggests long-term adaptations within the NE circuitry. We therefore decided to examine the expression of genes critical to NE signaling and regulation in this developmental group and in the reference adult cohort. We found that mRNA expression of LC α2-A (ADRA2A), DBH and NET were all decreased in the P10–P21 CNO-treated animals relative to their vehicle controls. No such changes were seen in the P56–P67 group (ADRA2A: treatment F_(1,16)_ = 7.152, p = 0.02; post hoc: P10–P21, p = 0.01; P56–67, p = 0.73: DBH: treatment F_(1,16)_ = 4.907, P = 0.04; post hoc P10–P21, p = 0.01; P56–P67, p = 0.97: NET treatment F_(1,16)_ = 6.487, p = 0.02; post hoc P10–P21, p = 0.04; P56–P67, p = 0.46; n = 4–6/group) (Fig. 5A–C). Further, we detected no difference in either TH or MAO A mRNA expression in the LC in either cohort (Supplementary Fig. S5G,H).

We next examined expression levels of NE receptors in LC projection areas that receive extensive NE innervation and have been implicated in the behaviors tested in our earlier studies. Specifically, the prefrontal cortex (PFC), hippocampus, hypothalamus and amygdala were selected. We found that interfering with NE neuronal activity during P10–P21, but not during P56–67, resulted in significantly decreased expression of α2-A but not α1-A (ADRA1A) or α1-D (ADRA1D) in the PFC (α2-A : treatment F_(1,16)_ = 4.138, p = 0.06; post hoc P10–P21 p = 0.04; P56–67, p = 0.87; α1-A: treatment F_(1,16)_ = 0.4162, p = 0.53; post hoc P10–P21 p = 0.52; P56–67, p = 0.99; α1-D: treatment F_(1,16)_ = 1.699, p = 0.2; post hoc P10–P21 p = 0.11; P56–67, p = 0.99; n = 4–6/group) (Fig. 5D–F). In the hippocampus, α2-A and α1-A mean expression was lower in the CNO treated group compared to controls, but this did not reach significance after correction for multiple comparison (α2-A : treatment F_(1,16)_ = 2.569, p = 0.013; post hoc P10–P21 p = 0.07; P56–67, p = 0.99; α1-A: treatment age × treatment interaction F_(1,16)_ = 5.06, p = 0.04; post hoc P10–P21 p = 0.06; P56–67, p = 0.62; n = 4–6/group) (Fig. 5G–I). In contrast, the expression of these receptors appeared unchanged in the hypothalamus and amygdala for both the P10–P21 and the P56–67 cohorts (Supplementary Fig. S5A–F). Thus, changes to key components of the NE circuit were evident in select target areas in P10–P21 adult animals, but not in animals treated in adulthood.

Having observed altered mRNA expression of NE receptors, we assessed NE levels in the same brain regions in mice that were exposed to the P10–P21 intervention. We micro-dissected the LC, the PFC, the hippocampus, the hypothalamus and the amygdala to measure total NE content in each region by HPLC. Interestingly, NE content was decreased in the PFC of P10–P21 CNO-treated when compared to vehicle-treated mice (Fig. 5K) but not in the LC, hippocampus, hypothalamus or amygdala (Fig. 5J,L–N) (treatment W(_9,15,30_) = 2.514, p = 0.05; post hoc Dunnets T3 LC p = 0.99, PFC p = 0.03, HP p = 0.95, Hyp p = 0.58, Amy p = 0.99; n = 5/group). In addition, no differences were detected in serotonin or dopamine levels in these brain regions (Figure S6F-O).

### Administration of the α2-A receptor agonist, guanfacine, during p10–P21 phenocopies the adult behavioral effects of suppressing NE neuron activity at that time

One important milestone in the development of NE circuits is the rapid rise of firing rates and the establishment of α2A receptor mediated negative feedback during P10–P21 in the LC^16^. Given the extensive changes in α2-A receptor expression and function that we observed in the P10–P21 group treated with CNO we next tested whether direct stimulation of the α2-A receptor during this time would phenocopy our findings. Stimulation of α2-A receptors on noradrenergic neurons would act analogously to the hM4Di DREADD stimulation in these neurons (as they are both Gi coupled GPCR’s) with some additional post-synaptic effects. We decided to use guanfacine, an α2-A agonist that is routinely prescribed in children and adolescents to treat attention deficit hyperactivity disorder (ADHD)^34^.

**Figure 6:**
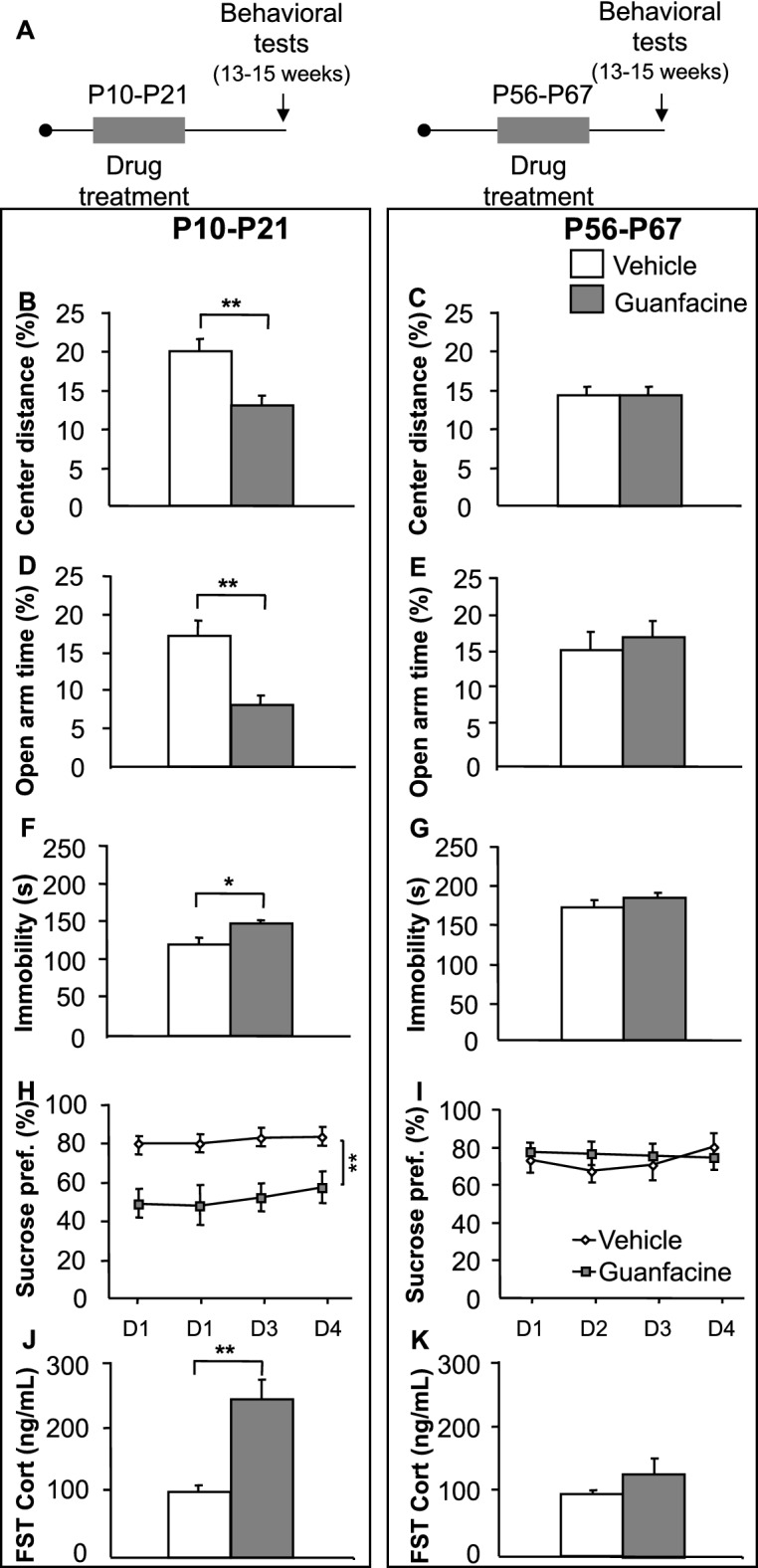
Guanfacine treatment between P10–P21, but not P56–P67, results in increased anxiety and depression-like behaviors in adulthood. (**A**) Experimental timeline. (**B**,**C**) P10–P21 guanfacine treatment, but not from P56–P67, results in decreased percent center distance in the open-field test, (**D**,**E**) and decreased percent time in the open arms in the elevated-plus maze test. (**F**,**G**) Increased immobility in the FST in the P10–P21 guanfacine group in adulthood when compared to their control mice. No such difference was observed in the P56–P67 group. (**H**,**I**) P10–P21 guanfacine administration, but not P56–P67, results in decreased sucrose preference. (**J**,**K**) Increased post forced swim stress-induced CORT release in P10–P21 guanfacine treated mice, but not in P56–P67, when compared to their respective controls. All means were compared using two-way ANOVA unless otherwise stated. Means are represented as ± SEM. (*p < 0.05; **p < 0.01). See also Supplementary Fig. S1B and S7.

Mice were treated with guanfacine (1 mg/kg) from either P10–P21 or P56–P67 (Fig. 6A and Supplementary Fig. S1B). Adult mice treated with guanfacine during P10–P21, but not those treated during P56–P67, displayed decreased percent center distance (treatment F_(1,64)_ = 5.991, p = 0.02, treatment × treatment age interaction F_(1,64)_ = 6.190, p = 0.02; post hoc P10–21 p < 0.001, P56-67 p = 0.99; n = 15–20/group) (Fig. 6B,C) along with no changes in the total path travelled in the open-field (Supplementary Fig. S7A,B). This is consistent with what was seen in the chemogenetic approach. However, we also found decreased percentage time in the open arms in the elevated-plus maze (treatment F_(1,64)_ = 3.416, p = 0.07; treatment age F_(1,64)_ = 4.763, p = 0.03; treatment × treatment age interaction F_(1,64)_ = 7.072, p = 0.01; post hoc P10–21 p < 0.001, P56–67 p = 0.83; n = 15–20/group) (Fig. 6D,E), a finding not detected with the chemogenetic approach and potentially attributable to actions of guanfacine at postsynaptic α2-A receptors.

Consistent with our chemogenetic approach results, we also observed increased immobility in the FST and decreased sucrose preference in mice that were treated with guanfacine during P10–P21, but not the adult period (FST: treatment F_(1,64)_ = 6.672, p = 0.01, treatment age F_(1,64)_ = 23.25, p < 0.001; post hoc P10–21 p = 0.04, P56–67 p = 0.35; Sucrose Preference: treatment F_(1,54)_ = 4.734, p = 0.03, treatment age F_(1,54)_ = 1.829, p = 0.18; treatment × treatment age interaction F_(1,54)_ = 7.192, p = 0.01 post hoc P10–21 p < 0.01, P56–67 p = 0.93; n = 15–20/group) (Fig. 6F–I). Moreover, the P10–P21 group also showed an increased CORT response after a forced-swim stressor (treatment F_(1,35)_ = 9.175, p < 0.001; treatment age F_(1,35)_ = 17.64, p < 0.001; treatment × treatment age interaction F_(1,35)_ = 7.785, p = 0.001; post hoc P10–21 p < 0.001, P56–67 p = 0.55; n = 15–20/group) (Fig. 6J,K). Finally, clonidine induced changes in body temperature in adulthood were attenuated in P10–P21 mice but not in P56–67 mice (Supplementary Fig. S7C,D). Thus, treatment of pups from P10–P21 with the α2-A agonist guanfacine broadly reproduces the effects seen from chemogenetically suppressing NE neuronal function during this time period.

## Discussion

Our results demonstrate the existence of a developmental sensitive period between P10 and P21, during which disruption of NE signaling leads to enduring adult behavioral phenotypes relevant to depression and anxiety. While our chemogenetic and pharmacological approaches cannot distinguish between effects on peripheral vs central adrenergic neurons, nor between distinct populations of central adrenergic neurons, we have focused our analysis on the LC effects, the most studied adrenergic nucleus and the one hypothesized accounted for the behavioral differences seen as a result of the intervention. The P10–P21 period is characterized by emergence of spontaneous NE activity and a potent α2-A autoreceptor mediated negative feedback in the LC^16^. This finding is in contrast to findings during an earlier NE sensitive period (P2–P9) when NE signaling is critical for developing attachment behavior^13,14,16^. The attachment sensitive period closes as α2-A spontaneous activity and feedback emerges at P10. We did not identify long-lasting effects of a P2–P9 DREADD intervention on adult behavioral phenotypes relevant to depression and anxiety.

While disrupting of NE activity had a lasting effect in our studies, NET disruption during a similar time-period (P4–P21) does not have long-term effects on similar behaviors^35^. Our results together with the earlier studies support a hypothesis in which NE neuronal activity during this sensitive period rather than NET function, is critical for establishing autoreceptor mediated feedback. Hence, low levels of activity during this time result in decreased α2A autoreceptor activity throughout life.

Once autoreceptor feedback is established, NE levels continue to steadily increase until plateauing near adult levels in early adolescence^16^. Our findings that after P33, interfering with NE firing no longer had enduring sequelae suggest that in the context of stabilizing NE levels, homeostatic setpoints for NE circuit function are more resistant to disruption. Taken together, our data suggest that there is a complex interplay between the emergence of spontaneous NE neuronal activity, the establishment of negative feedback and regional baseline NE levels, which ultimately results in determining adult emotion-like behaviors.

Although the noradrenergic system has been historically classified via anatomically defined nuclei, recent evidence suggests that these nuclei have developmentally heterogeneous origins, and that their function might be a result of their rhombomeric origin rather than just anatomic location^1^. As our interventions cannot directly attribute our results to specific nuclei or rhombomeric origin, we focused our studies on the LC as it is a major source of NE in the forebrain. Molecular interrogation of NE machinery in the P10–P21 cohort revealed distinct alterations in the LC and its target regions. The LC was broadly affected including reduced mRNA levels for the rate limiting synthesis enzyme DBH, the reuptake transporter NET, and the inhibitory α2-A autoreceptor responsible for feedback inhibition. In this regard, the absence of change in basal NE levels in the LC raises the possibility that interfering with LC firing during the sensitive period changes LC ability to mount stress responses. Our results indicating that fewer LC TH neurons express c-fos following stress, support this possibility. Furthermore, a change in LC reactivity would be expected to be accompanied by molecular adaptations in NE target regions. Indeed, molecular NE machinery in some LC target regions, such as the PFC, but not others was permanently altered.

As a result of disrupting NE signaling during P10–P21, we found reductions in α2-A receptor expression in the hippocampus and PFC but not in the hypothalamus or amygdala. Interestingly, these regions receive their primary NE innervation from distinct populations of NE neurons, with the hippocampus and PFC receiving greater innervation from the rhombomere 1 derived LC and the hypothalamus and amygdala receiving greater innervation from rhombomere 4 derived neurons in other NE nuclei. Since all the NE-producing nuclei were inhibited with our intervention, a provocative and testable possibility is that the P10–P21 sensitive period for NE modulation is specific to the LC, or to rhombomere 1 derived neurons^1,36–38^.

Interestingly, only the PFC exhibited a long-lasting decrease in baseline NE suggesting a shift in a homeostatic set point for NE signaling. While it is difficult to infer how different NE levels in the PFC may translate to behaviors, PFC neuronal activity controls behavior in the FST and modulates response to reward^39,40^. Together the results highlight circuit specific changes that are associated with depression-related but also with some anxiety-related features following inhibition of NE function during a sensitive period.

NE-acting medications are part of the pharmacological arsenal used to treat psychiatric disorders in children and their use has been increasing^41^. In particular, guanfacine has been used for treating ADHD^42^ and clonidine for post-traumatic stress disorder^43^. While the sensitive period identified here in mice is likely more relevant to the last trimester of pregnancy/the toddler years in humans, our studies highlight the critical need to fully understand the implications of treating developing brains with pharmacologic agents targeting modulatory amines. There has been relatively little research on drug by development interactions but given the potential long-term consequences of treatments during sensitive periods, additional consideration by physicians prescribing psychoactive medications to young children seems warranted.

## Materials and methods

### Animal husbandry

Animals were housed in groups of three to five per cage and had ad libitum access to food and water. Animals were maintained on a 12:12 light/dark schedule (6 a.m.–6 p.m.); all testing was conducted during the light period. Animal protocols were approved by the Institutional Animal Care and Use Committee and were conducted in accordance to the NIH Guide for the Care and Use of Laboratory Animals and in compliance with the ARRIVE guidelines. Care was taken to minimize the number of animals used and their suffering.

### Generation of DBH-hM4Di mice

RC::PDi (Cre-dependent inhibitory DREADD [hM4Di] receptor) mice have been described^28^ and were a generous gift from Susan Dymecki. Tg(Dbh-cre)KH212Gsat/Mmcd. (DBH-Cre) mice, identification number 032081-UCD, were obtained from the Mutant Mouse Regional Resource Center, a NCRR-NIH funded strain repository, and was donated to the MMRRC by the NINDS funded GENSAT BAC transgenic project. RC::PDi and DBH-Cre lines were crossed to generate the DBH-Cre-RC::PDi line (DBH-hM4Di +), which was maintained on a mixed C57BL/6-129S6/Sv background.

### Drug treatment and administration

*Clozapine-N-oxide (CNO)*: CNO was obtained from the NIH as part of the Rapid Access to Investigative Drug Program. For adult acute administration, CNO was injected at a dose of 5 mg/kg in 1% DMSO in 0.9% saline intraperitoneally (i.p.).

For developmental interventions, DBH-hM4Di^+^ male mice were treated daily with vehicle or CNO i.p. (5 mg/kg in 1% DMSO and 0.9% saline) during different time windows (P2–P21/P2–P9/P10–P21/P33–P44/P56–P67). Specifically, 10 μL per 1 g of mouse body weight was injected from a 0.5 mg/mL stock solution. For pre-weaning treatments (P21), the entire litters were removed from dams and placed in a small tray containing bedding from the respective home cage. The tray was placed on a scale allowing us to measure the weight of an individual pup when removing it for injection. Mice were injected in a random order and immediately placed back in the home cage. All mice within a litter were assigned to the same treatment.

*Clonidine induced hypothermia*: For systemically induced hypothermia, clonidine (0.5 mg/kg in 0.9% saline) and 8-OH-DPAT (1 mg/kg in 0.9% saline) (Sigma–Aldrich, St. Louis, MO)^44^ were administered i.p. For local injections, 1 μL of Clonidine (2 μg/μL) was infused bilaterally into the locus coeruleus (LC) via an internal infusion needle inserted into a guide cannula connected to a microsyringe (Hamilton, Reno, NV).

*Guanfacine*: Male mice were treated daily with vehicle or Guanfacine i.p. (1 mg/kg/day in 0.9% saline) during two distinct time windows (P10–P21 and P56–P67). Specifically, 10 μL per 1 gr of mouse body weight was injected from a 0.1 mg/mL stock solution. Pre-weaning treatments (P10–P21) were performed as in our chemogenetic intervention.

### Behavioral and physiological studies

Behavioral and physiological testing was performed over 4–5 weeks starting at 13–15-week old age (Supplementary Fig. S1).

### Open-field test

Exploration of a novel open field was measured for 30 min as previously described^45,46^. The center of the arena was defined as a square area occupying the center 50% of the total arena. Dependent measures were total path length (cm) and percent distance in the center (distance travelled in the center divided by total distance travelled).

### Elevated-plus maze

Animals were placed into the central area facing one open arm and allowed to explore the maze for 5 min as previously described^45,46^. Recorded videos were analyzed with TopScan software (Clever Sys Inc, Reston,VA). Dependent measures were time in the open arms and percent time in the open arms (time in the open arms divided by the total time).

### Sucrose preference

An 8-day sucrose preference protocol divided into 4 training and 4 testing days was performed as previously described^45,46^. On days 1 and 2 of training, mice were presented with two water filled bottles (water, water) for 2 h and 1 h respectively. On days 3 and 4 of training, both bottles contained 1% sucrose in water (sucrose, sucrose) for 1 h and 30 min respectively. On choice days 5–8 (testing days 1–4), one bottle contained water and the other 1% sucrose solution for 30 min each day (water, sucrose). Daily preference was calculated as: ((weight bottle 1)/(weight bottles (1 + 2)) × 100).

### Forced-swim test

Mice were placed into clear plastic buckets 20 cm in diameter and 23 cm deep filled 2/3 of the way with 26 °C water and videotaped from the side for 6 min^45,46^. This was done on two consecutive days. The last 4 min of each day were scored. Scoring was done using an automated Viewpoint Videotrack software package (Montreal, Canada), which was validated before by manual scoring. Dependent variable was immobility averaged across the two days.

### CNO and clonidine induced hypothermia

Mice were singly housed in clean cages for an hour and three baseline temperatures were taken (− 60 min, − 30 min, 0 min). Immediately after the last baseline measurement, animals received CNO (for validation of the system) or clonidine (to assess α2-A receptor function) as described above. Body temperature was assessed rectally using a lubricated probe (Thermalert TH-5 thermal monitor, Physitemp, Clifton, NJ) every 10 min for 60 or 120 min. Data in Fig. 1C is based on average drop in temperature across 60 min.

### Corticosterone measurements

Blood was collected during the dark–light transition and during the light–dark transition as previously described^45,46^. For stress-evoked corticosterone, experiments were performed starting at 12.00. Mice were exposed to a forced swim stressor for 6 min and blood was drawn from the submandibular vein 12 min later. Blood was centrifuged and plasma was isolated and stored until processed. Corticosterone levels were assessed by ELISA (Enzo Life Sciences, Farmingdale, NY)^45,46^.

### C-fos immunohistochemistry

C-fos was induced as previously described by a forced swim stressor^45^. After tissue processing, LC sections were stained using rabbit anti-c-fos antibody (1:5000, Millipore) and sheep anti-tyrosine hydroxylase (TH) (1:1000, Abcam) as previously described^45^.

### Quantitative PCR

Total RNA was extracted using TRIzol and SuperScript^®^ III First-Strand Synthesis System was used to synthesize cDNA, and PCR was performed and quantified using SYBR Green real-time PCR Master Mix (Life Technologies, Grand Island, NY). Primers used in the real-time qPCR are in the supplementary information.

### Statistical analysis and reliability

All statistical analyses were performed using Prism 9 (Graphpad). Final group numbers are shown in the figure legends. The results were expressed as mean ± SEM. *p* ≤ 0.05 was used as the threshold for significance. Group differences were analyzed using one or two-way analysis of variance (ANOVA) as appropriate, with Sidak post hocs unless otherwise stated. Welch ANOVA was used when significant differences in sample variance existed. Dunnet T3 post hocs were used in this case. Mixed model ANOVA was used for hypothermia and sucrose preference experiments. Drug treatments were assigned randomly by cage. The experimenter was blind to the treatment condition of the subjects at the time of behavioral testing. To further minimize experimenter bias, where possible, automated scoring of tests was conducted as described. Sample sizes for experiments were chosen based on past experience with the behavioral tests conducted.

## Supplementary Information

Supplementary Information.

## Data Availability

The datasets used and/or analyzed during the current study available from the corresponding author on reasonable request.
