# Supplementary material for: Inhibition of norepinephrine signaling during a sensitive period disrupts locus coeruleus circuitry and emotional behaviors in adulthood
Source: Sci Rep. 2023 Feb 22;13:3077. doi: 10.1038/s41598-023-29175-x (PMC9946949; doi:10.1038/s41598-023-29175-x)
Supplement: Supplementary file 1 — Supplementary Information. [file 41598_2023_29175_MOESM1_ESM.docx]

**SUPPLEMENTARY INFORMATION**

**Inhibition of norepinephrine signaling during a sensitive period disrupts locus coeruleus circuitry and emotional behaviors in adulthood.**

**Qingyuan Meng*, Alvaro L. Garcia-Garcia*, Alex Dranovsky and E. David Leonardo.**

******

***Figure S1. Related to Figure 2, 3 and 5. Timeline experimental cohorts of mice.* (A)** Cohorts used for pharmacogenetic manipulations of NE signaling during different time windows. **(B)** Cohorts used for guanfacine administration during different time windows. OF: Open Field, EPM: Elevated Plus Maze, Suc Pref: Sucrose Preference, FST: Forced Swim Test, AM-PM CORT: dark-light transition and light-dark transition corticosterone levels, FST ind CORT: forced swim induced corticosterone. Procedures are listed in the order in which they were administered with 2-3 days in between experiments.

**TH+ cells**

DBH-hM4Di^-^

DBH-hM4Di^+^

**B**

P7-P9

**A**

**C-fos+ cells**

******

0

200

400

600

800

P7-P9

0

500

1000

1500

2000

***Figure S2. Related to Figure 1. Pharmacogenetic inhibition of NE neurons at P7-P9.* (A)** Decrease in the number of c-fos+ cells (T-test p=0.01) along with **(B)** no changes in the number of TH+ cells (T-test p=0.76) (n=3-4/group). Means are represented as ±SEM. (*p<0.05; **p<0.01).

******

***Figure S3. Related to Figure 2. Pharmacogenetic inhibition of NE signaling during development does not impact locomotor activity or baseline corticosterone levels in adult mice*. (A-D)** No changes were detected in total path travelled in open-field in adult mice after CNO treatment during different time windows (main effect of treatment F_(1,131)_=0.003, p=0.95; treatment age F_(3,131)_=16.96, p<0.001; treatment X treatment age interaction F_(3,131)_=1.073, p=0.36); n=15-19/group). **(E-H)** No significant difference between Vehicle vs CNO treated groups in corticosterone levels at the onset of both the light (AM) and the dark phase (PM) at any of the ages tested (Treatment P2-P9: F_(1,32)_=0.005, p=0.94; AM: p>0.99; PM: p=0.99; treatment P10-P21 F_(1,32)_=0.036, p=0.85; AM: p=0.99; PM: p=0.97; treatment P35-P44: F_(1,32)_=0.015, p=0.90; AM: p>0.99; PM: p=0.98 P56-P67: F_(1,30)_=0.017, p=0.90; AM: p=0.98; PM: p=0.92; n=9-10/group). Means are represented as ±SEM.


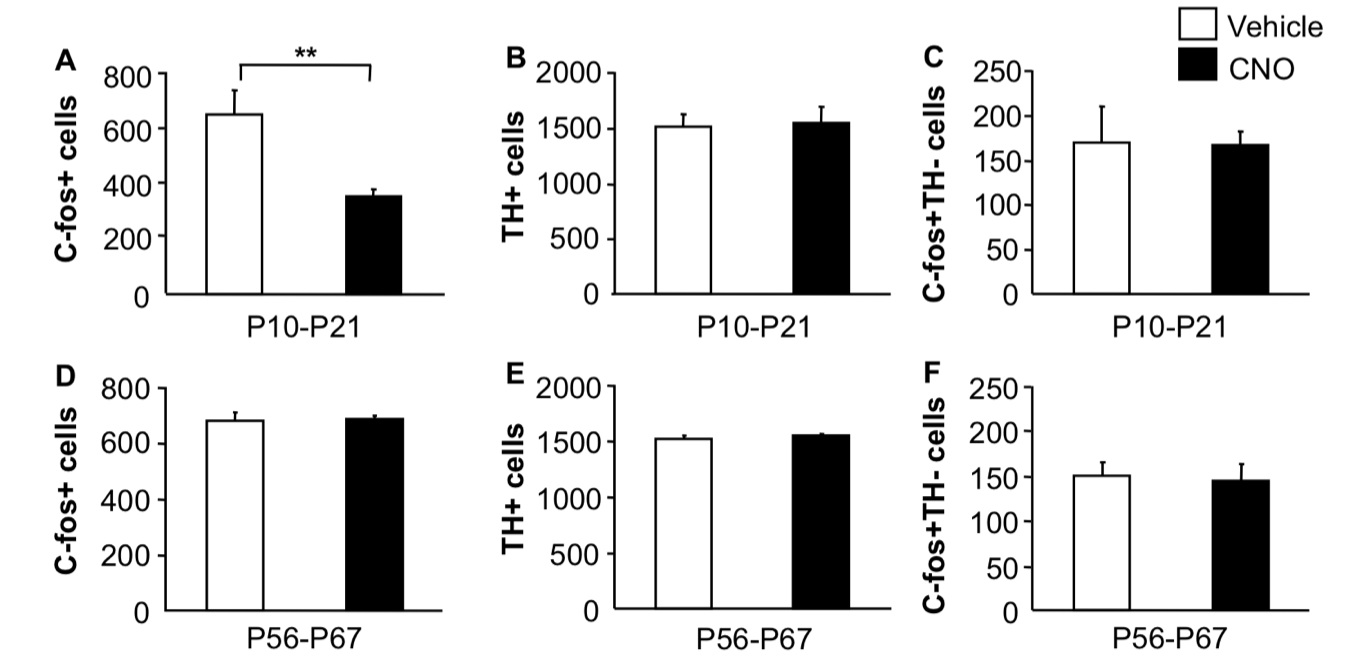


***Figure S4. Related to Figure 4. Pharmacogenetic inhibition of NE neurons between P10-P21, but not P56-P67, alters the LC response to stress in adulthood.* (A**,**D)** P10-P21 NE inhibition, but not P56-P67 results in a decreased number of stress induced c-fos+ cells (treatment F_(1,15)_=6.963, p=0.02; treatment age F_(1,15)_==1.169, p=0.30; treatment x treatment age interaction F_(1,15)_==10.37, p=0.0057; post hoc P10-P21 p<0.01; P56-P67 p=0.90), **(B,E)** but does not affect the number of NE (TH+) neurons (treatment F_(1,15)_==0.072, p=0.79; treatment age F_(1,15)_==0.21, P=0.65) **(C,F)** or the number of c-fos+TH- cells (treatment: F_(1,15)_=<0.001, p=0.99; treatment age F_(1,15)_==0.5366, p=0.48) in the LC of adult mice after P56-P67 CNO treatment of DBH-hM4Di^+^ mice (n=4-5/group for all measures). Means are represented as ±SEM. (*p<0.05; **p<0.01).


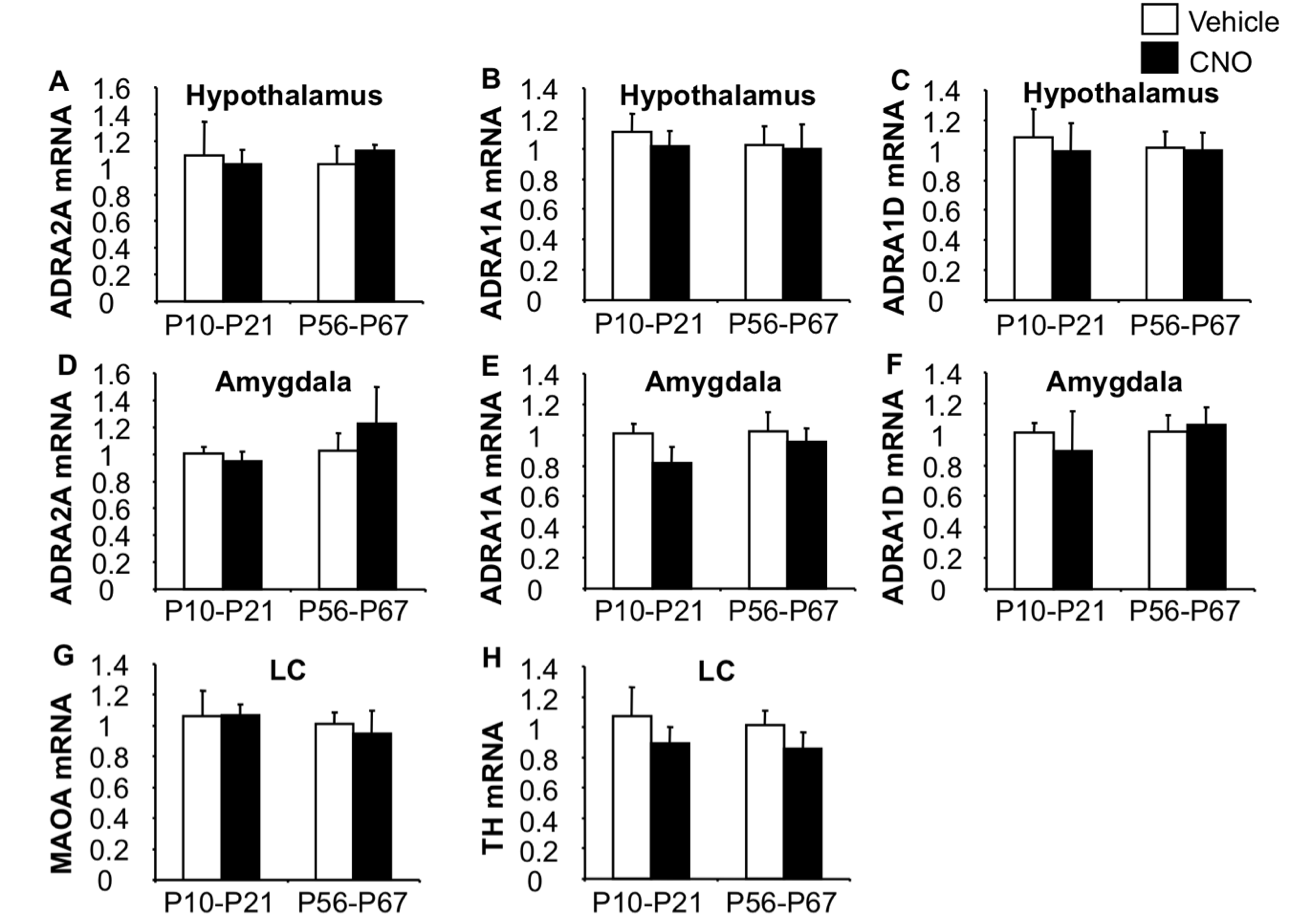


***Figure S5*. *Related to Figure 5.*  Lack of LC-NE system adaptations after pharmacogenetic inhibition of NE neurons*.* (A-C)** No changes were observed in the hypothalamus after P10-P21 or P56-P67 intervention in ADRA2A (treatment F_(1,16)_=0.008, p=0.93; treatment age F_(1,16)_=0.014, p=0.91; treatment age x age interaction F_(1,16)_=0.2731, p=0.61), ADRA1A (treatment F_(1,16)_=0.2042, p=0.66; treatment age F_(1,16)_=0.154, p=0.70; treatment age x age interaction F_(1,16)_=0.066, p=0.80) or ADRA1D mRNA levels (treatment F_(1,16)_=0.117, p=0.74; treatment age F_(1,16)_=0.04, p=0.84; treatment age x age interaction F_(1,16)_=0.2731, p=0.83). **(D-F)** No changes were observed in the amygdala after P10-P21 or P56-P67 intervention in ADRA2A (treatment F_(1,16)_=0.22, p=0.64; treatment age F_(1,16)_=0.94, p=0.35; treatment age x age interaction F_(1,16)_=0.69, p=0.42), ADRA1A (treatment F_(1,16)_=1.693, p=0.21; treatment age F_(1,16)_=0.60, p=0.45; treatment age x age interaction F_(1,16)_=0.422, p=0.53)or ADRA1D mRNA levels (treatment F_(1,16)_=0.05, p=0.83; treatment age F_(1,16)_=0.254, p=0.62; treatment age x age interaction F_(1,16)_=0.2063, p=0.66). **(G)** In the LC, no changes were observed in adult MAO A (treatment F_(1,16)_=0.026, p=0.87; treatment age F_(1,16)_=0.536, p=0.47; treatment age x age interaction F_(1,16)_=0.075, p=0.79) or **(H)** TH (Tyrosine hydroxylase) (treatment F_(1,16)_=1.553, p=0.23; treatment age F_(1,16)_=0.122, p=0.73; treatment age x age interaction F_(1,16)_=0.008, p=0.93) mRNA levels after P10-P21 and P56-P67 NE signaling inhibition. (n=4-6/group for all measures). Means are represented as ±SEM. (*p<0.05; **p<0.01).

***Figure S6*. *Related to Figure 5*. Early life pharmacogenetic inhibition of NE neurons only impacts PFC NE levels in adulthood. (A-E)** Levels of NE varied across tissue, but P10-P21 pharmacogenetic inhibition results in decreased norepinephrine (NE) levels in the PFC without changes in the LC, HP, HYP or Amygdala (treatment W(_9,15,30_)=2.514, p=0.05; post hoc Dunnets T3 LC p=0.99, PFC p=0.03, HP p=0.95, Hyp p=0.58, Amy p=0.99). Similarly, DA and 5-HT levels varied across tissues, but there was no significant difference in dopamine levels (treatment W(_9,15.7_)=9.45, p<0.0001; post hoc Dunnets T3 LC p=0..28, PFC p=0..99, HP p=0..81, Hyp p=0..74, Amy p=0..99) (**F-J**) or in serotonin levels (treatment W(_9,16.23_)=10.74, p<0.0001; post hoc Dunnets T3 LC p=0.72, PFC p=0..99, HP p=0..94, Hyp p=0..75, Amy p=0.59) between CNO and vehicle treated animals. (Locus coeruleus (LC), prefrontal cortex (PFC), hippocampus (HP), hypothalamus (HYP) amygdala (AMY) (n=5/per group). Means are represented as ±SEM. (*p<0.05) Welch ANOVA followed by pairwise comparison with Dunnets T3.


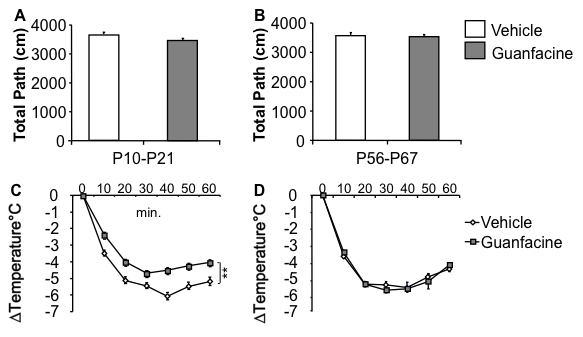


***Figure S7. Related to Figure 6. Guanfacine administration during P10-P21 or P56-P67 developmental time windows does not impact locomotor activity in adulthood, but administration of Guanfacine during P10-P21 impacts clonidine induced hypothermia in adult mice.*** No changes were detected in total path travelled in open-field in adult mice that were treated with guanfacine during **(A)** P10-P21 (main effect of treatment age_(F1,56)_=; post hoc P10-21 p=0.18, P56-67 p=0.86). **(C, D)** Guanfacine from P10 to P21, but not from P56 to P67, leads to a decreased hypothermic response to Clonidine in adulthood (main effect of treatment F_(1,8)_=33.11, p<0.001; time F_(6,48)_=731.4, p<0.001; time X treatment age interaction F_(6,48)_=12.95, p<0.001). (n=10/group for A, B and 5/group for C, D). Means are represented as ±SEM. (*p<0.05; **p<0.01).

**SUPPLEMENTARY MATERIALS AND METHODS**

*Drugs treatment and administration*

Clozapine-N-oxide (CNO) was obtained from the NIH as part of the Rapid Access to Investigative Drug Program funded by the NINDS. It was dissolved in 1% DMSO and 0.9% Saline.

For hypothermia in figure 1 CNO was injected at a dose of 5 mg/kg in vehicle (0.9% Saline with 1% DMSO) intraperitoneally (i.p.) in adulthood.

For developmental interventions, DBH-hM4Di+ male mice were treated daily with vehicle or CNO i.p. (5 mg/kg in 1% DMSO and 0.9% saline) during different time windows (P2–P9/ P10-P21/ P33-P44/ P56-P67). Specifically, 10 μL per 1 gr of mouse body weight was injected from a 0.5 mg/mL stock solution. For preweaning treatments (P21), the entire litters were removed from dams and placed in a small tray containing bedding from the respective home cage. The tray placed on a scale allow us to measure the weight of an individual pup when removing it for injection. Mice were injected in a random order and immediately placed back in the home cage. Because we aimed at keeping a minimal interference within the litters we assigned all mice within a litter to the same treatment. All mice were housed in the same room and rack and were weaned on P21 and housed in groups of five mice.

For clonidine-induced hypothermia, clonidine (0.5 mg/kg in 0.9% saline)(Sigma–Aldrich, St. Louis, MO, USA) was administered i.p. For 8-OH-DPAT induced hypothermia, 1mg/kg in 0.9% saline (Sigma–Aldrich, St. Louis, MO, USA) was injected i.p. 8-OH-DPAT induced [hypothermia](http://topics.sciencedirect.com/topics/page/Hypothermia) in mice is dependent on functional 5-HT_1A_ autoreceptors (Richardson-Jones *et al*, 2011).

Guanfacine was dissolved in vehicle (0.9% saline). Male mice were treated daily (3-5 pm) with vehicle or Guanfacine i.p. (1 mg/kg/day in 0.9% saline) during different time windows (P10-P21/ P56-P67). Specifically, 10 μL per 1 gr of mouse body weight was injected from a 0.1 mg/mL stock solution. Pre-weaning treatments (P10-P21) were performed as in our pharmacogenetic intervention.

*Behavioral and physiological studies*

All animals used for behavioral testing were age matched within 2 weeks. Male mice were tested starting at 13–15-week old age. Behavioral and physiological testing was performed over 4–5 weeks in the following order: open-field, elevated-plus maze, sucrose preference and forced swim test and hypothermia with a minimum of 2-3 days between each test. All behavioral testing took place during the light cycle.

*Immunohistochemistry*

*P7-P9 pups c-fos studies*

The entire litter of P7-P9 DBH-hm4Di (-) and (+) pups was removed from dams and placed in a small tray containing bedding from the respective home cage. The tray placed on a scale allow us to measure the weight of an individual pup when removing it for CNO (5 mg/Kg) injection. Mice were injected in a random order and immediately placed back in the home cage. Mice were anesthetized and transcardially perfused 90 minutes later.

*Tissue processing*

After anesthesia with ketamine and xylazine (100 mg/ml ketamine; 20 mg/ml xylazine), mice were perfused transcardially (cold 0.1 M phosphate buffer (pH = 7.4) (PBS) followed by 4% paraformaldehyde (PFA). Brains were removed, post-fixed (24 h), cryoprotected in a 30% sucrose solution (in phosphate buffer) and stored at 4 °C. Serial sections (35 μM) were cut through the entire brain on cryostat (Leica CM3050 S) and stored in PBS with 0.1% NaN3. Specifically, sections at 1:6 interval trough the LC were used.

*Immunofluorescence studies*

For visualization purposes, free floating coronal serial sections (35 μm) of the LC were first washed with PBS 3x10 min followed by 30 min incubation with Triton 1% in PBS. Afterwards, sections were blocked in 10% NDS for 1 hour at room temperature and incubated in primary antibodies for 72 hrs at 4°C (1:100, rabbit anti-HA (Invitrogen, Camarillo, CA), 1:1000, sheep anti-tyrosine hydroxylase (TH) (Abcam)). After washing with PBS, sections were incubated for 1 hr with the secondary antibody donkey anti-rabbit biotin (1:200)(Jackson ImmunoResearch, West Grove, PA)) followed by amplification with avidin (1:200, Cy3 (Jackson ImmunoResearch, West Grove, PA)) complex and donkey anti-sheep cy2 (1:200) (Jackson ImmunoResearch, West Grove, PA) and NeuroTrace fluorescent Nissl stain (Invitrogen, Grand Island, NY). Thereafter, sections were washed 2x10min followed by a last 30 min. wash and mounted on glass slides and embedded with Prolong Gold Antifade Reagent (Invitrogen, Grand Island, NY).

For the stress-induced c-fos experiments, c-fos was induced as previously described by a forced swim stressor (10 min) and mice were perfused 2 hours after stress (Garcia-Garcia *et al*, 2013). After tissue processing, LC sections were washed with PBS 3x10min followed by incubation with Triton 1% in PBS for 30 min. Afterwards, serial sections were blocked in 10% Normal donkey serum (NDS) and 1% Triton in PBS for 1 hr. and then incubated with rabbit c-fos antibody (1:5000, Millipore) and sheep TH (1:1000, Abcam) overnight at 4 °C. After washing with PBS, sections were incubated for 1 hr with secondary antibody (1:200 donkey anti-rabbit biotin (Jackson ImmunoResearch, West Grove, PA)) followed by amplification with avidin (1:200, Cy3 (Jackson ImmunoResearch, West Grove, PA)) complex and donkey anti-sheep (1:200, Cy2 (Jackson ImmunoResearch, West Grove, PA). Thereafter, sections were washed 2x10min followed by a last 30 min. wash and mounted on glass slides and embedded with Prolong Gold Antifade Reagent (Invitrogen, Grand Island, NY).

*Image processing and quantification*

For all experiments, sections were imaged with identical exposure times, and parameters with a confocal microscope (Leica, NY, USA) at a magnification of 20x. 4-5 LC sections per mouse were counted. Each section was assessed for the number of single c-fos+ and TH+ cells as well as the number of double-labeled cells. An investigator blind to the conditions counted only very intense c-fos red stained cells. C-fos red signal what was surrounded by green (TH) was counted as double positive. Further, co-localization of induced c-fos immunoreactivity with TH cell bodies was confirmed with a stack analysis of the images and evaluating the sections in Z series (ImageJ software).

*Quantitative PCR*

Brains were removed from the mice after cervical dislocation. Brain regions were dissected under microcope and storage at -80ºC. Total [RNA](http://www.sciencedirect.com/science/article/pii/S0306452215005278#200002070) from the tissues was extracted using TRIzol (Life Technologies, Grand Island, NY, USA). The SuperScript® III First-Strand Synthesis System (Life Technologies, Grand Island, NY, USA) was used to synthesize cDNA, and [PCR](http://www.sciencedirect.com/science/article/pii/S0306452215005278#200021912) was performed and quantified using SYBR Green [real-time PCR](http://www.sciencedirect.com/science/article/pii/S0306452215005278#200024504) Master Mix (Life Technologies, Grand Island, NY, USA). Analysis was performed for Adrenergicα2A receptor (ADRA2A), Adrenergicα1A receptor (ADRA1A), Adrenergicα1D receptor (ADRA1D), monoamine oxidase A (MAOA) and TH mRNA expression. The β-actin mRNA expression was analyzed as the internal control. Primers used in the real-time quantitative PCR were shown as follows. For ADRA2A, the sense primer was 5'-CTGGACACGGACCTGCTT-3' and the antisense primer was 5'-GAGGCTTCATTTCCTTCTGC-3'. For ADRA1A, the sense primer was 5'-TCAATGAGGAGCCAGGATA-3' and the antisense primer was 5'-GATACGGAGCGTCACTTGC-3'. For ADRA1D, the sense primer was 5'-GTGTCCAGCCTGTCCCATAA-3' and the antisense primer was 5'-CGTCTTGGGGAACATTTAGG-3'. For MAOA, the sense primer was 5'-CTCGGATATTCTCAGTCACCA-3' and the antisense primer was 5'-GAGGACCATTATCTGTTCACTTATT-3'. For TH, the sense primer was 5'-GTCTACTGTCTGCCCGTGAT-3' and the antisense primer was 5'-CAATGTCCTGGGAGAACTGG-3'. For GAPDH the sense primer was 5’-GCCTTCCGTGTTCCTACCC-3’ and the antisense primer was 5’-TGAAGTCGCAGGAGACAACC-3’. For β-actin, the sense primer was 5’-GACGGCCAGGTCATCACTAT-3’ and the antisense primer was 5’-ATGCCACAGGATTCCATACC-3’.

*Brain neurotransmitter levels*

NE, DA and 5-HT concentrations were determined by HPLC as previously described (Garcia-Garcia et al, 2015). Specifically, all assays were carried out on a Waters Xevo TQ MS ACQUITY UPLC system (Waters, Milford, MA, USA). Concentrations of compounds in the samples were quantified by comparing integrated peak areas against those of known amounts of purified standards. Loss during extraction was accounted for by adjusting for the recovery of the internal standard added before extraction. The results were normalized by sample weight.

*Statistical Analysis*

All statistical analyses were performed using Stat View (SAS Institute Inc.). Final group numbers are shown in Figure legends. Results from data analyses were expressed as mean ± SEM. *p*<0.05 was used as the threshold for significance. Group differences were analyzed using a one-way analysis of variance (ANOVA) unless otherwise stated. Mixed measures ANOVA was used for hypothermia and sucrose preference experiments.

**SUPPLEMENTAL REFERENCES**

Garcia-Garcia AL, Meng Q, Richardson-Jones J, Dranovsky A, Leonardo ED (2015). Disruption of 5-HT function in adolescence but not early adulthood leads to sustained increases of anxiety. Neuroscience.

Garcia-Garcia AL, Venzala E, Elizalde N, Ramirez MJ, Urbiola A, Del Rio J, et al (2013). Regulation of serotonin (5-HT) function by a VGLUT1 dependent glutamate pathway. Neuropharmacology 70: 190-199.

Richardson-Jones JW, Craige CP, Nguyen TH, Kung HF, Gardier AM, Dranovsky A, et al (2011). Serotonin-1A autoreceptors are necessary and sufficient for the normal formation of circuits underlying innate anxiety. J Neurosci 31(16): 6008-6018.
